# Presence of an acute phase response in sheep with clinical classical scrapie

**DOI:** 10.1186/1746-6148-8-113

**Published:** 2012-07-17

**Authors:** Siv Meling, Kjetil Bårdsen, Martha J Ulvund

**Affiliations:** 1 Norwegian School of Veterinary Science, Department of Production Animal Clinical Sciences, Section for Small Ruminant Research, Kyrkjevegen 332-334, N-4325, Sandnes, Norway

## Abstract

**Background:**

Work with experimental scrapie in sheep has been performed on-site for many years including studies on PrP^Sc^ dissemination and histopathology of organs and tissues both at preclinical and clinical stages. In this work serum was sampled at regular intervals from lambs which were infected immediately after birth and from parallel healthy controls, and examined for acute phase proteins. In contrast to earlier experiments, which extensively studied PrP^Sc^ dissemination and histopathology in peripheral tissues and brain, this experiment is focusing on examination of serum for non-PrP^Sc^ markers that discriminates the two groups, and give insight into other on-going processes detectable in serum samples.

**Results:**

There was clear evidence of an acute phase response in sheep with clinical scrapie, both experimental and natural. All the three proteins, ceruloplasmin, haptoglobin and serum amyloid A, were increased at the clinical stage of scrapie.

**Conclusion:**

There was evidence of a systemic measurable acute phase response at the clinical terminal end-stage of classical scrapie.

## Background

Scrapie is a fatal neurodegenerative disease of sheep, and is one of the diseases in the group called Transmissible Spongiform Encephalopathies (TSEs), where PrP^Sc^, an abnormal form of the normal cellular PrP^C^, is believed to be the infective agent [1]. Other TSEs are Creutzfeldt-Jakob disease (CJD) in man, Bovine Spongiform Encephalopathy (BSE) in cattle and Chronic Wasting Disease (CWD) in deer, to mention a few. TSEs were at first regarded as neurodegenerative diseases without an inflammatory component, but the characterisation of a marked functional activation of microglial cells and identification of increased cytokine expression in the affected areas of the brain, points towards a localised inflammatory response [2]. The PrP^Sc^ and its dissemination and related histopathology have been, and is still, extensively studied in several different animal models, especially in murine models. Scrapie affected sheep (both experimentally and naturally infected) with the most susceptible PrP genotype, VRQ/VRQ and VRQ/ARQ, have a preclinical PrP^Sc^ dissemination in peripheral lymphoid tissue throughout the body, and this has proven useful in preclinical diagnosis by immunohistochemistry (IHC) and Western Blot (WB) of sheep of such PrP genotypes [3-8]. Less susceptible PrP genotypes have shown to have a less predictable lymphatic tissue involvement, thus increasing the risk of false negative lymphoid biopsies [9,10]. During the last decade there has been an increasing interest in the search for other non-PrP^Sc^ disease related biomarkers for TSEs by the use of different -omics techniques, especially in the search for preclinical diagnostic markers that could be used for diagnostics in the live animal. In the search for non-PrP^Sc^ markers of scrapie much work has been done in screening mostly brain, but also peripheral lymphoid tissue and serum in different murine models, for different gene expressions, especially different cytokines which have been detected both at mRNA and protein levels [11-16]. Not all of these murine models show the same results, and Tribouillard-Tanvier et al (2009) suggest that this may be attributable to the animal model used [15]. Recently, Huzarewich et al (2010) published a review on different potential disease markers detected in the application of -omics to prion biomarker discovery [17]. In the search for a non-PrP^Sc^ marker of prion diseases it is quite noticeable that many of the significant genes and proteins are linked to the activation of microglia in the brain, and the processes in and around the innate immune response including the acute phase response.

Measurements of serum or plasma proteins in relation to diagnostics have been performed for many years, and particularly acute phase proteins (APPs), which increase or decrease in response to a number of inflammatory insults as part of the acute phase response (APR) [18]. The APR is part of the innate immune system and first line of defence. It is the organism’s first response to tissue injuries, infections, stress and inflammation, and is characterised by a local reaction at site of injury followed by a number of systemic reactions including changes in the concentration of acute phase proteins (APPs) [19-21]. The majority of serum proteins are synthesised in the liver, including the APPs, which are part of the APR. The APR is thought to be beneficial to the animal in restoring homeostasis, and the response is tightly controlled by negative feedback loops, as the APR itself can cause harm if it comes out of control.

Measurements of APPs in serum/plasma can be used to assess the innate immune system’s systemic response to infection, inflammation, trauma and other pathological injuries; they have even been suggested as markers for overall herd/flock health in farm animals by detecting subclinical conditions. The APPs are mainly synthesised in hepatocytes, but also extrahepatically, and they are induced by cytokines including interleukin-1 (IL-1), interleukin-6 (IL-6) and tumor-necrosis factor- alpha (TNF-α) released mainly from macrophages, monocytes and astrocytes at site of lesion [22,23]. Measurements of APPs have shown to be useful as diagnostic and prognostic markers in several conditions in addition to assessing response to treatment, as their levels correspond to tissue damage. APPs lack specificity, but are highly sensitive indicators of inflammation and tissue injury [24].

Serum amyloid A (SAA), haptoglobin (Hp) and Ceruloplasmin (Cp) have been described as important APPs in sheep, with SAA and Hp being the major APPs in this animal species [22,23,25]. Increased plasma and serum levels of these proteins have been associated with a variety of diseases and conditions, and neither SAA nor Hp are usually detected at all in healthy sheep. Hp and Cp have been shown to increase significantly in experimental *Mannheimia haemolytica* infection, and remain elevated for ten days before returning to pre-infection levels [26]. Cp is an effective antioxidant, also in the CNS, protecting neural cells from oxidative stress, which plays a crucial role during CNS injury, when free iron and reactive oxygen species (ROS) increase [27].

Moderate increases in SAA and Hp have been detected in subclinical infections, and, on this basis, it has been suggested that measurements of these two APPs can become useful in determining health status in flocks. As there is an association between APP levels and the severity of disease it has also been proposed that APPs can be used as prognostic indicators [25,26,28-33].

This work is part of a larger study to investigate non-PrP^Sc^ markers in serum from experimentally infected sheep. In previous work by Ersdal et al. the inoculation was performed between 46 and 61 days of age, while this later model, described by Ulvund et al, the lambs are inoculated at birth [34,35]. This later model has a relatively short incubation period where the animals show clinical disease already at 4–5 months of age, and the distribution of PrP^Sc^ is extensive in the brain at clinical end stage. Initial proteomic analysis of serum from these sheep at the terminal clinical end stage revealed serum amyloid A (SAA) levels in the scrapie group to be significantly differently expressed from the healthy group, and by latent variable methods, was found to discriminate affected from healthy sheep with 95% correct classification rate (data not shown). To our knowledge, this represents the first verification of a measurable APR in sheep with both experimental and naturally occurring classical scrapie. The APR was mainly measured by Hp, SAA and Cp levels in serum.

## Results

### Experimental classical scrapie model

This experimental model of classical scrapie has been reported elsewhere [34,35]. All the animals in the scrapie group developed clinical signs of classical scrapie within time of euthanasia at 23 weeks of age/post infection. Already at four months of age, very subtle clinical signs of pruritus and wool eating were detected; though only on video surveillance. Clear clinical signs of disease were first obvious during the last week before euthanasia, and clinical presentation deteriorated quickly with noticeable pruritus, wool changes, ataxia, depressed mental status and recumbency. Very few macroscopic changes were recorded during post mortem examination, apart from redness and abrasions of skin in areas of pruritus and wool loss. The brain from all the animals in both groups were examined for PrP^Sc^ by immunohistochemistry (IHC) and WB, and only the animals in the scrapie group were positive (data not shown).

### Total protein and albumin measurements and globulin calculation

The mean total protein (TP) concentration in both groups increased steadily from six weeks of age (woa) until a relatively high peak at 18 woa (control group, mean ± SEM, 79.5 ± 0.87 mg/ml) and at 20 woa (scrapie group at 80.2 ± 1.91 mg/ml). TP level in the control group then decreased to reach 68.1 ± 1.71 mg/ml at 24 woa. TP level in the scrapie group also declined after peaking, but later increased again at euthanasia at 23 woa (77.2 ± 3.53 mg/ml), creating a significant difference between the groups (p < 0.05). This significant difference in TP level was in majority due to the higher globulin level in the scrapie group (49.9 ± 2.3 mg/ml) compared to the control group (40.2 ±1.5 mg/ml).

**Figure 1:**
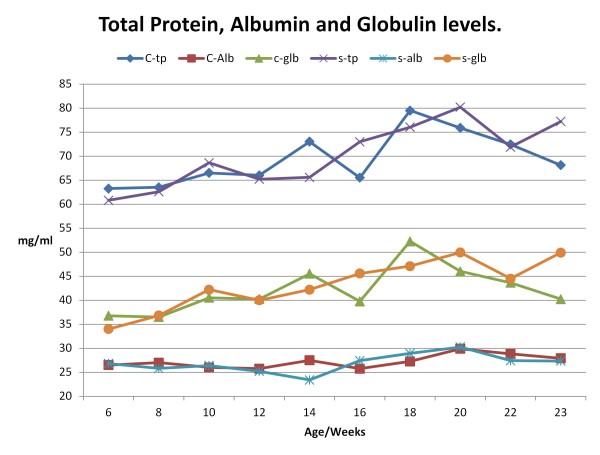
**Serum levels of total protein, Albumin and calculated Globulin in both groups from 6 to 23/24 weeks of age.** Total protein level in control group (♦ blue) and scrapie group (× purple), Globulin levels for control group (▴ green) and scrapie group (● orange), and albumin levels for control group (■ red) and scrapie group (∗ turquoise). Each time point is presented as the mean values for each group at each time of sampling.

Globulin levels in both groups increased and decreased in the same fashion as TP levels, and the fluctuation in TP levels seemed to be the result of globulin changes. This was not the case at 14 woa, where there was a significant difference in TP levels between the groups (p < 0.05). Coinciding with this, there was a significant decrease in albumin concentration (p < 0.05) in the scrapie group (23.4 ± 0.8 mg/ml) compared to the control group (27.5 ± 0.9 mg/ml). Changes in these proteins over time are presented in Figure 1.

### Acute phase protein responses in experimental classical scrapie

SAA and Hp concentrations were from undetectable to very low in the majority of samples during the study. Analytical sensitivity of the two kits used was 0.3 μg/ml for SAA and 0.005 mg/ml for Hp (according to the manufacturer). One control animal had detectable SAA response at ages 10, 12 and 18 woa, and increased Hp measurements at 10 and 14 woa. Two scrapie animals had increased levels of both SAA and Hp at 10 and 14 woa respectively. No clinical signs of disease were detectable at any of these incidences in either group. From 20 woa, SAA and Hp levels increased in the scrapie group, while remained undetectable in the control group. Measurements in serum from one of the animals in the scrapie group had only a moderate level of SAA (2.69 μg/ml) at 22 weeks and undetectable level of end stage, and Hp measurements at the same times where at the levels measured for the control group.

**Figure 2:**
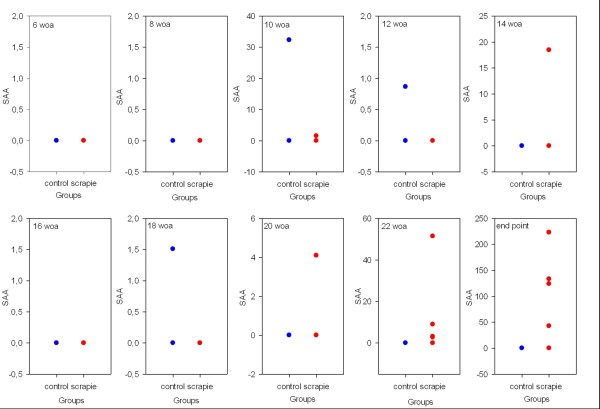
**Comparison of difference in SAA levels in serum between healthy controls and scrapie infected sheep from six to 23/24 weeks of age.** Each of the individual five scrapie samples (red dots) and four control samples (blue dots) are presented individually as a single dot to indicate the measured serum amyloid A (SAA) level which is presented on y-axis in μg/ml. At most time points, individual samples in both groups were below the detection range of the SAA kit, and thus the dots appear on top of each other as one single dot at 0.0 μg/ml.

The four animals in the control group were sampled ten times each, and out of these 40 individual samples, only three had detectable SAA levels, and all the three samples were from the same animal (ranged 0 to 32.3 μg/ml). Of the 50 samples from the scrapie group, 39 had undetectable SAA levels, two animals had isolated incidences of elevated SAA levels at a subclinical stage of scrapie at 10 (1.54 μg/ml) and 14 (18.46 μg/ml) woa. From 20 weeks of age, there was a tendency for SAA levels to increase more dramatically until time of euthanasia (ranged 0 to 222.8 μg/ml). The individual SAA measurements are shown in dot plots representing each time point sampled in Figure 2.

**Figure 3:**
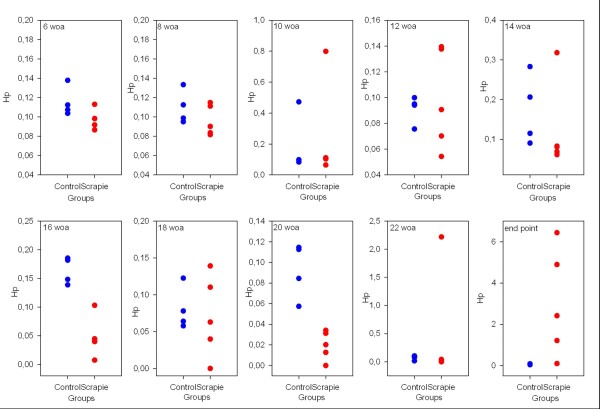
**Comparison of difference in Hp levels in serum between healthy controls and scrapie infected sheep from six to 23/24 weeks of age.** Scrapie (red dots), controls (blue dots), haptoglobin (Hp) levels measured in mg/ml indicated on y-axis and groups are presented on the x-axis. Each sample is individually plotted (four control samples and five scrapie samples), but, at some time points, the dots appear as one single measurement due to very similar values and the scale accommodates the scrapie measurements, as they show more variation and a higher levels.

Hp levels in the control group were low throughout the sampling period with a mean value of 0.11 ± 0.01 mg/ml, and the mean level in the scrapie group up until 22 weeks of age was similar at 0.10 ± 0.02 mg/ml. Thereafter the haptoglobin level increased steeply to reach a mean value of 3.0 ±1.17 mg/ml. Hp measurements are presented individually in a dot plot at each time point in Figure 3.

**Figure 4:**
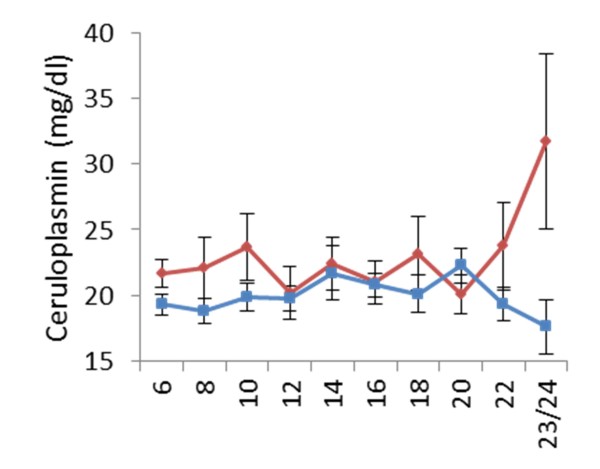
**Comparison of ceruloplasmin (Cp) between healthy controls and scrapie infected sheep from six to 23/24 weeks of age.** Scrapie (♦ red dotted line), controls (■ blue), Ceruloplasmin (Cp).

Cp levels were slightly higher in the scrapie group throughout most of the studied period, but the most prominent difference was detectable from 22 weeks of age and onwards. During the last fortnight, the Cp levels in the control group decreased, while there was a sharp increase in the scrapie group, resulting in a significant difference (p < 0.05) between the two groups at time of euthanasia (31.7 ± 6.7 vs. 17.6 ± 2.0). Figure 4.

### Natural cases of classical scrapie

All the natural cases of classical scrapie were confirmed by histopathological examination of the brain by typical lesions as well as detection of PrP^Sc^[3,35]. Four out of these five animals had shown pruritus for more than three weeks, two of these developed severe neurological signs before euthanasia, and one animal was without any visible clinical signs (Table 1). TP levels in these five sheep ranged from 57.6 to 93.8 mg/ml (70.9 ± 7.1 mg/ml). Albumin levels were relatively stable for all five sheep at 22.6 ± 0.8 mg/ml, and slightly lower in one sheep (19.6 mg/ml). Calculated globulin levels ranged from 34.5 to 70.1 mg/ml (48.2 ± 7.3 mg/ml). Hp concentration was compared to the experimental control group and not found to be statistically different (p > 0.05). One animal had a very high level of Hp at 7.15 mg/ml, while the remaining four had a mean of 0.18 mg/ml. All the animals had detectable SAA levels, ranged from 0.43 to 273.3 μg/ml, with significant difference from the control group (p < 0.05). Results for all the measured serum proteins are presented in Table 2.

**Table 1:** Presentation of the natural cases of classical scrapie

**ID**	**Admitted**	**Sampled**	**Age**	**Genotype**	**Clinical presentation**	**Brain**	**Brain PrP**^**Sc**^
S1	10.02.94	14.02.94	6	VRQ/VRQ	Pruritic, nervous, tremors	Vacuolisation with astrocytosis	Positive
S2	20.03.96	07.05.96	5	VRQ/VRQ	Pruritic, ataxia, nervous, altered mental state	Vacuolisation with astrocytosis	Positive
S3	27.08.98	16.09.98	7	ARQ/VRQ	Pruritus with hyperkeratosis	Vacuolisation with astrocytosis	Positive
S4	14.07.99	15.09.99	2	ARQ/VRQ	Normal	Vacuolisation with astrocytosis	Positive
S5	14.02.01	23.02.01	7	VRQ/VRQ	Pruritic	Vacuolisation with astrocytosis	Positive

Overview over admission dates and time of euthanasia, age in years, PrP genotype, clinical presentation at time of euthanasia and pathognomonic histopathology of the brain of the five natural classical scrapie cases. PrP^Sc^ was identified by immunohistochemistry on sections of the brain as described by Ersdal et al. (2005) [3]

**Table 2:** TP, Alb, Glb, Hp and SAA measurements in the five natural classical scrapie cases at time of euthanasia

**ID**	**Tp (mg/ml)**	**Alb (mg/ml)**	**Glb (mg/ml)**	**Hp (mg/ml)**	**SAA μg/ml**
S1	64.2	23.7	40.5	0.29	273.30
S2	80.5	19.6	60.9	7.15	1.31
S3	93.8	23.7	70.1	0.18	8.55
S4	58.2	23.7	34.5	0.16	0.43
S5	57.6	22.5	35.1	0.07	0.43
C				0.06	-

Level of different proteins in serum from five cases of naturally occurring classical scrapie. Hp and SAA levels were compared to measurements of the oldest animals in the control group. The “-“ indicates that measured levels were below detection range of test.

### Evaluation Hp and SAA kit performance

The coefficients of variation for both kits are presented in Table 3. Intra-assay CV for Hp and SAA was 4.6% and 10.6%, respectively, and inter-assay CV for Hp and SAA was 0.9% and 9.1%. The limitations in these measurements were that the inter-assay CV was based on only two separate plates and the first plate mean was calculated from only one duplicate. The second mean was calculated from the same duplicate samples as used for intra-assay CV. To achieve a better estimate of the inter-assay CV, more samples and assays would be needed. The intra-assay CV was based on similar numbers of samples as presented by the manufacturer.

**Table 3:** Intra- and inter-assay coefficient of variation of Hp and SAA kits

	**Intra-assay CV**		**Inter-assay CV**		
	**n**	**Average % CV**	**Mean of means**	**SD of means**	**% CV of means**
Hp	7	4.6	11.62	0.10	0.9
SAA	13	10.6	22.28	2.04	9.2

CV: Coefficient of variation; n: number of duplicate samples; SD: Standard Deviation; Hp: haptoglobin; SAA: Serum amyloid A.

## Discussion

In this work, we describe increased levels of certain APPs in sheep with experimental and natural cases of clinical classical scrapie. Such rise of these APPs in sheep has not earlier been published in classical scrapie. Regarding protein degradation during storage, serum samples from experimental scrapie had been stored in aliquots at −70 °C, and the sera from natural cases had been stored at −20 °C. All the samples had been stored for more than one year, and one of the natural cases was from 1994. The deteriorating effects on protein concentration in these samples are unknown, but it has been shown that albumin and total proteins levels remain stable for at least eight months in canine serum at −20 °C [36] and recently Gislefoss et al. reported non-significant differences of albumin in samples stored for 25 years, two years and one month [37]. Based on these examinations, the results achieved in this work were regarded to be reliable and indicative of the true protein status in the samples. Cp measurements were based on its oxidative activity, which decreases over time in storage, and therefore Cp was not measured in the naturally occurring classical scrapie cases.

As age has a major effect on the concentration of many serum proteins, it was difficult to find reference ranges based on animals in the age range of 6 weeks to six months of age [17,38-41]. Total serum protein has a wide dynamic range, thereby making analysis difficult to interpret when comparing to reference ranges developed by others. In addition, the protein levels may also be influenced by breed, management systems and analytical methods, and available reference ranges usually do not give either of these influential factors. Therefore, it may be misleading to uncritically compare results with any reference range listed. The animals used in this project were kept under very strict management system, and all the animals were managed similarly, they are of the same breed and age. Due to reasons mentioned above, the serum proteins were compared between the groups in question at same age, breed and feeding regime and management.

The total protein and albumin profile changed over time in a similar fashion to what was described by Kaneko [42], although the total protein levels in both groups were slightly to moderately higher than those reported for lambs younger than 12 months. Albumin levels were clearly lower. As albumin and globulins make up most of the total serum protein, globulin levels were higher than indicated by others [38-41]. There was a general increase in total protein from birth, combined with a minimal change in albumin and a marked increase in globulins with advancing age, reaching high levels at 18 – 20 weeks of age. At birth, the total protein is relatively low, but increase rapidly after ingestion of colostrum and maternal immunoglobulins. As globulins declined due to normal turnover, the animals begin to synthesize their own immunoglobulins, and reach adult levels of albumins and globulins in young adulthood. Total protein levels reached above average adult levels as a result of a minor decrease in albumin and progressive increase in globulins [42]. From 20 weeks of age, there were significant differences in TP levels between the groups, with a marked increase in the scrapie group. This increase was mainly due to a significant increase in the globulin fraction. As the APPs and immunoglobulins are part of the globulin fraction, this increase could be the result of increased synthesis of APPs, as seen for the three positive APPs tested in this work: Hp, SAA and Cp. Several studies have failed to describe a significant increase in immunoglobulins at clinical classical scrapie, especially scrapie-specific IgG [43-45].

There are very few available reference ranges for APPs in sheep, but recently Lepherd *et al.*[39] published good reference ranges for SAA and Hp in young sheep. Although these reference intervals were from a different breed and sheep kept under different management systems, they were in the same age range and seemed appropriate in that manner. Skinner and Roberts [32] evaluated Hp levels in a variety of conditions, both infectious and non-infectious, in addition to healthy sheep, and concluded that Hp values above 0.2 g/l were “positive”. It is also interesting to see that 7% of the randomly selected healthy sheep had a positive Hp value, which could indicate the presence of subclinical conditions. In other studies, levels of SAA below 2 μg/ml (range 0 to 29.4) have been reported in clinical healthy sheep [33,39]. APPs are often reported as undetectable to very low levels in healthy individuals, and subclinical conditions would often lead to a temporary elevation before decreasing to resting levels. Thus the moderate increase in SAA and Hp seen approximately half-way through the incubation period in two animals in the scrapie group, and one control, could be due to a subclinical condition. As no clinical signs of disease were detected in any of the animals at this time, it is difficult to rule in or out any specific conditions, including scrapie. There is a possibility that the increase in SAA and Hp in the two scrapie infected animals at 10 and 14 weeks of age could relate to the expected PrP^Sc^ dissemination through peripheral lymphoid tissue that is seen about half-way through the incubation period in this experimental model. This would need further testing to confirm. Batxelli-Molina et al. (2010) reported transthyretin to discriminate scrapie affected sheep from healthy sheep, both during early asymptomatic phase and later during the symptomatic phase, although only significant at the late stage [46]. Transthyretin is a negative APP, and thus decreased levels would be an expected finding along with increased levels of positive APPs during an APR. The clear and significant increase in SAA, Hp and Cp measured at the clinical end stage indicate a detectable APR at this stage of classical scrapie, both in the experimental and the natural cases. This coincides well with the pathological changes seen on histopathological examination of the brain and the onset of severe clinical disease seen at this late stage in this experimental model. APPs levels are reported to be directly related to severity of both tissue damage and level of inflammation, i.e. they are specific markers for tissue damage and inflammation [25]. Pro-inflammatory cytokines, like interleukin 6 (IL-6), interleukin-1 (IL-1) and tumor-necrosis factor α (TNF-α), are released by damaged tissues locally and into the circulation, where more inflammatory cells are activated. These responses result in production of more cytokines and inflammatory mediators circulating in the blood acting on different target organs/cells leading up to a systemic reaction with alteration in synthesis of APPs in the liver and activation of the hypothalamic-pituitary-adrenal (HPA) axis [30]. This stimulation will lead to increased production of cortisol by the adrenal glands, which has a negative feedback effect on this axis. This stimulation of the HPA axis could contribute to the hypercorticism with increased cortisol, 20β-dihydrocortisol and cortisone detected in plasma and urine of sheep with subclinical and clinical scrapie that has been reported with a certain discriminating level [47-49]. Cytokines induce production of adrenocorticotrophic hormone (ACTH) which in turn stimulates synthesis of the corticosteroid, cortisol [50,51]. This is an important regulatory feature in inflammatory and cytokine responses, as adrenalectomized rodents show increased mortality in experiments involving injection of bacterial lipopolysaccharide, IL-1 or TNF-α. After administration of glucocorticoids, these mice survived. Thus by regulating cytokine production and action, HPA axis contributes to modulation of the inflammatory response [52].

SAA and Hp are characterised as major acute phase proteins (APPs) in sheep and these are known to increase with severity of tissue damage and level of inflammation. Interestingly, the SAA response in these experimental cases of scrapie are higher than levels reported for mulesing and caseous lymphadenitis (CLA) [29,31], and more in the region of experimental mastitis [33]. It is tempting to think that the APP levels in the naturally affected animals were related to extent of histopathological changes in the brain, as the two animals with clear neurological signs had highest levels of APP. The others with much lower levels of APP presented with pruritus as the main clinical sign, although all of them had pathological changes in the brain. It is also worth noticing the clear increase in the globulin fraction of the TP in three out of the five natural cases which was outside the reference range [42]. Relating these results to clinical findings, these three sheep were presented with the most severe clinical signs. Albumin level in the worst clinically affected sheep were even lower than the others, perhaps due to the on-going APR. This leads to the thought that the systemic effects of scrapie was related to severity of clinical signs and could be measured in serum by SAA, Hp and Cp levels. Measuring APP levels in serum may provide objective information about the extent of the on-going process, magnitude and duration may reflect the severity [22]. Even though the APR is a non-specific response, it is a feature of clinical scrapie, and due to the high sensitivity of the APR, measurements of APPs could be useful in evaluation of the underlying pathological processes and the systemic involvement of scrapie.

APPs respond rapidly to insults and the majority peak about one day after the initial insult before returning to resting levels within a week due to the fact that most APPs have half-life of only 24 to 48 hours [25]. This means that some important time points may have been missed. Even though this experimental model gives fairly predictive and consistent development of classical scrapie and clinical signs at around the same time, individual differences may result in individual APP expression profiles. A different sampling strategy with more frequent blood sampling could detect more subtle changes in the APP profile of scrapie affected sheep throughout the incubation period.

The exact underlying cause or the actual triggering factors behind the detectable increase in APPs seen in this study remains unknown, but this response coincides well with the known accumulation of PrP^Sc^ in the brain, astrocytosis and vacuolar changes seen at this late clinical stage in VRQ/VRQ animals [3,4,34]. Several murine models of classical scrapie show that pro-inflammatory cytokines like IL-1, IL-6 and TNF-α, have increased expression at both mRNA and protein levels in brain, peripheral lymphoid tissue and serum at the terminal end stage [11,12,14,15]. Microglia produce pro-inflammatory cytokines like IL-1, IL-6 and TNF-α upon activation, and these are stimulators of the production of most APPs [19,22,53]. Newsom et al. (2011) reported recently quite an extensive list of proteins with altered expression in brain, lymphoid tissue and serum both pre-clinically and clinically in a murine model [14]. Volkel et al. (2001) investigated plasma samples from patients with CJD and found significant increase in both C-Reactive Protein (CRP) and Interleukin-6 (IL-6) compared to healthy controls, and concluded that these could be assessed for cell damage and inflammation in similar fashion to other markers[54]. Coe et al. (2001) showed in an experimental scrapie murine model increasing plasma levels of serum amyloid P with the onset and progression of clinical signs [55]. There exist differences in expression patterns both in the brain and peripheral in the different murine models, and as postulated by Newsom et al, this could be attributable to the animal model used [14]. There are only a couple of records of APPs being of significance in scrapie in sheep, serum transthyretin and urinary α-1Antichymotrypsin have been found to discriminate healthy from scrapie infected both at pre-clinical and clinical stage of scrapie [46,56].

## Conclusion

Based on the results from these different murine models and the two reports on sheep, our results are of great relevance as we described increased levels of the major APPs in sheep which to our knowledge has not been published in relation to scrapie in sheep. It is also interesting to see that in an experimental model as the one used here, where PrP^Sc^ dissemination, pathological changes and onset of clinical disease are expected to show little between individual differences, there is a great variation in quantitative expression of the APPs.

Due to the low number of animals in this study and the lack of disease specificity of the acute phase response, these findings will need to be further evaluated in larger groups to establish the expected dynamic range of APPs in classical scrapie and other neurological and non-neurological diseases of sheep. Comparable conditions of interest are listeriosis, cerebrocortical necrosis, focal symmetrical encephalomalacia and tick born fever and encephalitis.

## Methods

### Experimental classical scrapie model

Nine Norwegian Rygja lambs with the same PrP genotype (homozygous V_136_R_154_Q_171_) were inoculated through a stomach tube with 1 gram of pooled brain material immediately after birth and before ingestion of colostrum as described by Ulvund et al [34]. Four lambs in the control group were inoculated with brain material originating from healthy scrapie-free sheep of the same genotype and PrP^Sc^ negative, and five lambs were inoculated with brain material originating from confirmed cases of classical scrapie. The lambs in both groups were kept with their dams for the whole time length of the project until euthanasia at 23–25 weeks of age. As the control group was born about two weeks before the animals in the scrapie group, animals in the control group were 25 weeks old at euthanasia, while the scrapie group were only 23 weeks old. Each dam and offspring were kept isolated in confined units without any contact with other animals, and the scrapie group was under video surveillance. Both groups were kept under similar conditions and feeding regimes. Animal experiments were approved by the Norwegian Animal Research Authority.

### Blood sample collection

Non-fasting blood samples were collected into 10 ml plain tubes (Terumo Venoject®) every fortnight from six woa until euthanasia at 23/25 weeks of age, for a total of ten times. Blood samples were allowed to clot at room temperature for a minimum of 30 minutes and maximum 60 minutes before processing. Serum was pipetted in aliquots and frozen at −80 °C within two hours of sampling. All the samples were subjected to the same handling procedures throughout the experiment. Serum was used for biochemistry, Hp and SAA concentration analysis.

### Natural cases of classical scrapie

During the early 1990s and until 2005, several cases of suspected classical scrapie from farms in the surrounding area were received at the Norwegian School of Veterinary Science, Section for Small Ruminant Research, for diagnostic purposes. The cases were admitted for clinical examination, blood sampling, genotyping and diagnosis. Sometimes these sheep were kept under video surveillance for some time while in isolation, before euthanasia and histopathological examination of the brain. Blood sampling was performed at least 48 hours after transport. Serum samples from five of these cases, stored at - 20 °C, were used for protein analysis. Table 1.

### Protein measurement

Serum total protein was measured with ABX Pentra Total Protein (TP), ceruloplasmin (CP) and albumin (Alb) with ABX Pentra Albumin CP, both kits from Horiba ABX Diagnostics (Montpellier, France). The oxidase activity of Cp in serum was determined using a colorimetric enzyme assay where oxidation of p-phenylenediamine dihydrochloride was measured and thereby estimating the Cp concentration in mg/dl. All three measurements were adapted for COBAS MIRA Plus equipment (Roche Diagnostics, Basel, Switzerland) and routinely performed in the laboratory.

Globulin concentration was calculated by subtracting albumin concentration from total protein measurement.

Serum Hp concentrations were determined manually using the Phase^™^ Haptoglobin Assay kit (Tridelta Development Limited, County Kildare, Ireland). All the samples were run in duplicates, and the mean of each duplicate was used to determine final concentration. The analyses were run according to manufacturer’s instructions and immediately read at 600 nm on a Multiskan* GO Microplate Spectrophotometer using SkanIt Software 3.2, both from Thermo Fisher Scientific (Waltham, MA, USA). Samples with signal greater than the highest standard were diluted and re-run until all the signals fell within the linear part of the standard curve.

SAA concentrations were determined using Phase™ Range Multispecies SAA ELISA kit (Tridelta Development Limited, County Kildare, Ireland). Sera were initially diluted 1:100 in Diluent buffer, and samples with signals greater than the highest standard were further diluted and re-run until all the signals fell within the linear part of the standard curve. Some samples were diluted 1:1000. The only automated part of the procedure was the washing steps, using the ELx50 Microplate Strip Washer (BioTek Instruments, Inc., Winooski, VT, USA). The absorbance was read on the Multiskan* GO Microplate Spectrophotometer using SkanIt Software 3.2 at 450 nm using 630 nm as a reference.

### Statistical analysis

The results were adjusted for age such that the groups were compared at the same animal age. At end point comparison, the lambs of the scrapie group were 23 weeks old and the control group was 24 weeks of age.

Significant difference (p < 0.05) of protein measurements between the two groups at each sampling was determined by calculation of the 95% confidence interval for the difference in population medians. A non-parametric test, Wilcoxon signed-rank test, was used for calculation of confidence interval and p-values, as normal distribution of data could not be assumed due to small sample size. SigmaPlot Version 12.0 (Systat Software Inc., Erkrath, Germany) and Microsoft Excel 2010 were used.

### Reference range used and evaluation of assay performance

As no reference ranges for any of the proteins evaluated here were available for Norwegian sheep breeds, and especially not for lambs in the age range of six to 24 weeks, the level of proteins in the scrapie group was evaluated in reference to the control group. The protein levels (TP, Alb and Glb) in the natural cases were evaluated in relation to reference ranges provided by Kaneko [42], this due to the great age difference to the experimental control group. HP and SAA levels were compared to the experimental control group at 24 woa.

Assay performance was evaluated by intra- and inter-assay coefficients of variation (CV). These were calculated by dividing the standard deviation by the mean of the same set of measurements and thereafter converting it into percentage. The intra-assay CV for SAA was calculated from 13 duplicate samples by averaging the CV (%) for each of the individual duplicates. Intra-assay CV for Hp was calculated in the same manner, based on seven duplicates. Inter-assay CV was calculated from the average CV value from two assays run six months apart. One assay was the same as used for intra-assay CV calculations and the other only contained one duplicate sample.

## Abbreviations

TSEs, Transmissible Spongiform Encephalopathies; PrPC, Normal Cellular Prion Protein; PrPSc, Scrapie Prion Protein; APP, Acute Phase Protein; APR, Acute Phase Response; TP, Total Protein; Alb, Albumin; Glb, Globulin; Cp, Ceruloplasmin; Hp, Haptoglobin; SAA, Serum Amyloid A; BSE, Bovine Spongiform Encephalopathy; CJD, Creutzfeldt-Jakob Disease; HPA, Hypothalamus-Pituitary-Adrenal; CLA, Caseous Lymphadenitis; IL, Interleukin; TNF, Tumor Necrosis Factor; ACTH, Adrenocorticotropic Hormone; ROS, Reactive Oxygen Species; woa, Weeks of age; IHC, Immunohistochemistry; WB, Western Blot; CV, Coefficient of variation; SD, Standard Deviation; SEM, Standard Error of Mean.

## Competing interests

The authors declare that they have no competing interests.

## Authors’ contributions

SM carried out the protein measurements, statistical data analysis and drafted the manuscript. KB participated in the design of the study, sample preparation and helped to draft the manuscript. MJU participated in the design and coordination of the study, sample collection and helped to draft the manuscript. All authors read and approved the final manuscript.

## References

[B1] Prusiner SB Novel proteinaceous infectious particles cause scrapie Science 1982 216 136 144 10.1126/science.6801762 6801762

[B2] Rezaie P Lantos PL Microglia and the pathogenesis of spongiform encephalopathies Brain Res Brain Res Rev 2001 35 55 72 11245886 10.1016/s0165-0173(01)00042-x

[B3] Ersdal C Ulvund MJ Espenes A Benestad SL Sarradin P Landsverk T Mapping PrPSc propagation in experimental and natural scrapie in sheep with different PrP genotypes Vet Pathol 2005 42 258 274 10.1354/vp.42-3-258 15872372

[B4] Ryder SJ Dexter GE Heasman L Warner R Moore SJ Accumulation and dissemination of prion protein in experimental sheep scrapie in the natural host BMC Vet Res 2009 5 9 10.1186/1746-6148-5-9 19243608 PMC2649917

[B5] Andreoletti O Berthon P Marc D Sarradin P Grosclaude J Van KL Schelcher F Elsen JM Lantier F Early accumulation of PrP(Sc) in gut-associated lymphoid and nervous tissues of susceptible sheep from a Romanov flock with natural scrapie J Gen Virol 2000 81 3115 3126 11086143 10.1099/0022-1317-81-12-3115

[B6] Espenes A Press CM Landsverk T Tranulis MA Aleksandersen M Gunnes G Benestad SL Fuglestveit R Ulvund MJ Detection of PrP(Sc) in rectal biopsy and necropsy samples from sheep with experimental scrapie J Comp Pathol 2006 134 115 125 10.1016/j.jcpa.2005.08.001 16466737

[B7] O'Rourke KI Baszler TV Besser TE Miller JM Cutlip RC Wells GA Ryder SJ Parish SM Hamir AN Cockett NE Preclinical diagnosis of scrapie by immunohistochemistry of third eyelid lymphoid tissue J Clin Microbiol 2000 38 3254 3259 10970367 10.1128/jcm.38.9.3254-3259.2000 PMC87369

[B8] Langeveld JP Jacobs JG Erkens JH Bossers A van Zijderveld FG van Keulen LJ Rapid and discriminatory diagnosis of scrapie and BSE in retro-pharyngeal lymph nodes of sheep BMC Vet Res 2006 2 19 10.1186/1746-6148-2-19 16764717 PMC1544330

[B9] Monleon E Garza MC Sarasa R Varez-Rodriguez J Bolea R Monzon M Vargas MA Badiola JJ Acin C An assessment of the efficiency of PrPsc detection in rectal mucosa and third-eyelid biopsies from animals infected with scrapie Vet Microbiol 2011 147 237 243 10.1016/j.vetmic.2010.06.028 20685048

[B10] Gonzalez L Dagleish MP Martin S Dexter G Steele P Finlayson J Jeffrey M Diagnosis of preclinical scrapie in live sheep by the immunohistochemical examination of rectal biopsies Vet Rec 2008 162 397 403 10.1136/vr.162.13.397 18375983

[B11] Campbell IL Eddleston M Kemper P Oldstone MB Hobbs MV Activation of cerebral cytokine gene expression and its correlation with onset of reactive astrocyte and acute-phase response gene expression in scrapie J Virol 1994 68 2383 2387 8139024 10.1128/jvi.68.4.2383-2387.1994 PMC236715

[B12] Cunningham C Wilcockson DC Boche D Perry VH Comparison of inflammatory and acute-phase responses in the brain and peripheral organs of the ME7 model of prion disease J Virol 2005 79 5174 5184 10.1128/JVI.79.8.5174-5184.2005 15795301 PMC1069550

[B13] Hwang D Lee IY Yoo H Gehlenborg N Cho JH Petritis B Baxter D Pitstick R Young R Spicer D A systems approach to prion disease Mol Syst Biol 2009 5 252 19308092 10.1038/msb.2009.10 PMC2671916

[B14] Newsom DM Liggitt HD O'Rourke K Zhuang D Schneider DA Harrington RD Cytokine antibody array analysis in brain and periphery of scrapie-infected Tg338 mice Comp Immunol Microbiol Infect Dis 2011 34 387 397 10.1016/j.cimid.2011.06.001 21788075

[B15] Tribouillard-Tanvier D Striebel JF Peterson KE Chesebro B Analysis of protein levels of 24 cytokines in scrapie agent-infected brain and glial cell cultures from mice differing in prion protein expression levels J Virol 2009 83 11244 11253 10.1128/JVI.01413-09 19710140 PMC2772806

[B16] Williams AE van Dam AM Man AHW Berkenbosch F Eikelenboom P Fraser H Cytokines, prostaglandins and lipocortin-1 are present in the brains of scrapie-infected mice Brain Res 1994 654 200 206 10.1016/0006-8993(94)90480-4 7987669

[B17] Huzarewich RL Siemens CG Booth SA Application of "omics" to prion biomarker discovery J Biomed Biotechnol 2010 2010 613504 20224650 10.1155/2010/613504 PMC2833310

[B18] Anderson NL Anderson NG The human plasma proteome: history, character, and diagnostic prospects Mol Cell Proteomics 2002 1 845 867 10.1074/mcp.R200007-MCP200 12488461

[B19] Gabay C Kushner I Acute-phase proteins and other systemic responses to inflammation N Engl J Med 1999 340 448 454 10.1056/NEJM199902113400607 9971870

[B20] Heinrich PC Castell JV Andus T Interleukin-6 and the acute phase response Biochem J 1990 265 621 636 1689567 10.1042/bj2650621 PMC1133681

[B21] Moshage H Cytokines and the hepatic acute phase response J Pathol 1997 181 257 266 10.1002/(SICI)1096-9896(199703)181:3<257::AID-PATH756>3.0.CO;2-U 9155709

[B22] Petersen HH Nielsen JP Heegaard PM Application of acute phase protein measurements in veterinary clinical chemistry Vet Res 2004 35 163 187 10.1051/vetres:2004002 15099494

[B23] Murata H Shimada N Yoshioka M Current research on acute phase proteins in veterinary diagnosis: an overview Vet J 2004 168 28 40 10.1016/S1090-0233(03)00119-9 15158206

[B24] Eckersall PD Bell R Acute phase proteins: Biomarkers of infection and inflammation in veterinary medicine Vet J 2010 185 23 27 10.1016/j.tvjl.2010.04.009 20621712

[B25] Cray C Zaias J Altman NH Acute phase response in animals: a review Comp Med 2009 59 517 526 20034426 PMC2798837

[B26] Ulutas PA Ozpinar A Effect of Mannheimia (Pasteurella) haemolytica infection on acute-phase proteins and some mineral levels in colostrum-breast milk-fed or colostrum-breast milk-deprived sheep Vet Res Commun 2006 30 485 495 10.1007/s11259-006-3246-z 16755360

[B27] Patel BN Dunn RJ Jeong SY Zhu Q Julien JP David S Ceruloplasmin regulates iron levels in the CNS and prevents free radical injury J Neurosci 2002 22 6578 6586 12151537 10.1523/JNEUROSCI.22-15-06578.2002 PMC6758125

[B28] Aziz DM Taha MB Effect of dystocia on serum haptoglobin in Awassi ewes Theriogenology 1997 48 559 562 10.1016/S0093-691X(97)00273-2 16728152

[B29] Eckersall PD Lawson FP Bence L Waterston MM Lang TL Donachie W Fontaine MC Acute phase protein response in an experimental model of ovine caseous lymphadenitis BMC Vet Res 2007 3 35 10.1186/1746-6148-3-35 18093286 PMC2235841

[B30] Gruys E Toussaint MJ Niewold TA Koopmans SJ Van DE Meloen RH Monitoring health by values of acute phase proteins Acta Histochem 2006 108 229 232 10.1016/j.acthis.2006.03.009 16714050

[B31] Lepherd ML Canfield PJ Hunt GB Thomson PC Bosward KL Assessment of the short-term systemic effect of and acute phase response to mulesing and other options for controlling breech flystrike in Merino lambs Aust Vet J 2011 89 19 26 21250952 10.1111/j.1751-0813.2010.00668.x

[B32] Skinner JG Roberts L Haptoglobin as an indicator of infection in sheep Vet Rec 1994 134 33 36 10.1136/vr.134.2.33 8135004

[B33] Winter P Fuchs K Walshe K Colditz IG Serum amyloid A in the serum and milk of ewes with mastitis induced experimentally with Staphylococcus epidermidis Vet Rec 2003 152 558 562 10.1136/vr.152.18.558 12751607

[B34] Ulvund MJ Bårdsen K Brundtland E Meling S Ersdal C Espenes A Press CM A New Experimental Model for Studying Scrapie and Prion Disease in Sheep Proceedings of the Prion2007 Conference: 26-28 September 2007 2007 Glasgow, Edinburgh 63

[B35] Ersdal C Natural and experimental scrapie in sheep: Studies on pathogenesis and morphology. PhD Thesis 2004

[B36] Thoresen SI Tverdal A Havre G Morberg H Effects of storage time and freezing temperature on clinical chemical parameters from canine serum and heparinized plasma Vet Clin Pathol 1995 24 129 133 10.1111/j.1939-165X.1995.tb00954.x 12664427

[B37] Gislefoss RE Grimsrud TK Morkrid L Stability of selected serum proteins after long-term storage in the Janus Serum Bank Clin Chem Lab Med 2009 47 596 603 19290843 10.1515/CCLM.2009.121

[B38] Alonso AJ de Teresa R Garcia M Gonzalez JR Vallejo M The effects of age and reproductive status on serum and blood parameters in merino breed sheep Zentralbl Veterinarmed A 1997 44 223 231 9270344 10.1111/j.1439-0442.1997.tb01104.x

[B39] Lepherd ML Canfield PJ Hunt GB Bosward KL Haematological, biochemical and selected acute phase protein reference intervals for weaned female Merino lambs Aust Vet J 2009 87 5 11 10.1111/j.1751-0813.2008.00382.x 19178470

[B40] Roubies N Panousis N Fytianou A Katsoulos PD Giadinis N Karatzias H Effects of age and reproductive stage on certain serum biochemical parameters of chios sheep under greek rearing conditions J Vet Med A Physiol Pathol Clin Med 2006 53 277 281 10.1111/j.1439-0442.2006.00832.x 16901268

[B41] Dubreuil P Arsenault J Belanger D Biochemical reference ranges for groups of ewes of different ages Vet Rec 2005 156 636 638 15894728 10.1136/vr.156.20.636

[B42] Kaneko JJ Kaneko JJ, Harvey JW, Bruss ML Serum Proteins and the Dysproteinemias Clinical Biochemistry of Domestic Animals 1997 Fifth Academic Press, San Diego 117 138

[B43] Sassa Y Kataoka N Inoshima Y Ishiguro N Anti-PrP antibodies detected at terminal stage of prion-affected mouse Cell Immunol 2010 263 212 218 10.1016/j.cellimm.2010.03.018 20417929

[B44] Strain GM Barta O Olcott BM Braun WF Serum and cerebrospinal fluid concentrations of immunoglobulin G in Suffolk sheep with scrapie Am J Vet Res 1984 45 1812 1813 6497137

[B45] Collis SC Kimberlin RH Further studies on changes in immunoglobulin G in the sera and CSF of Herdwick sheep with natural and experimental scrapie J Comp Pathol 1983 93 331 338 10.1016/0021-9975(83)90019-1 6863615

[B46] Batxelli-Molina I Salvetat N Andreoletti O Guerrier L Vicat G Molina F Mourton-Gilles C Ovine serum biomarkers of early and late phase scrapie BMC Vet Res 2010 6 49 10.1186/1746-6148-6-49 21044301 PMC2988006

[B47] Gayrard V Picard-Hagen N Grino M Sauze N Grandjean C Galea J Andreoletti O Schelcher F Toutain PL Major hypercorticism is an endocrine feature of ewes with naturally occurring scrapie Endocrinology 2000 141 988 994 10.1210/en.141.3.988 10698174

[B48] Picard-Hagen N Gayrard V Laroute V Grandjean C Andreoletti O Elsen JM Schelcher F Toutain PL Discriminant value of blood and urinary corticoids for the diagnosis of scrapie in live sheep Vet Rec 2002 150 680 684 10.1136/vr.150.22.680 12074236

[B49] Schelcher F Picard-Hagen N Laroute V Gayrard V Popot MA Andreoletti O Toutain PL Corticoid concentrations are increased in the plasma and urine of ewes with naturally occurring scrapie Endocrinology 1999 140 2422 2425 10.1210/en.140.5.2422 10218997

[B50] Johnson RW Inhibition of growth by pro-inflammatory cytokines: an integrated view J Anim Sci 1997 75 1244 1255 9159271 10.2527/1997.7551244x

[B51] Uhlar CM Whitehead AS Serum amyloid A, the major vertebrate acute-phase reactant Eur J Biochem 1999 265 501 523 10.1046/j.1432-1327.1999.00657.x 10504381

[B52] Bethin KE Vogt SK Muglia LJ Interleukin-6 is an essential, corticotropin-releasing hormone-independent stimulator of the adrenal axis during immune system activation Proc Natl Acad Sci USA 2000 97 9317 9322 10.1073/pnas.97.16.9317 10922080 PMC16865

[B53] Brown DR Microglia and prion disease Microsc Res Tech 2001 54 71 80 10.1002/jemt.1122 11455614

[B54] Volkel D Zimmermann K Zerr I Lindner T Bodemer M Poser S Schwarz HP C-reactive protein and IL-6: new marker proteins for the diagnosis of CJD in plasma? Transfusion 2001 41 1509 1514 10.1046/j.1537-2995.2001.41121509.x 11778065

[B55] Coe JE Race RE Ross MJ Serological evidence for an inflammatory response in murine scrapie J Infect Dis 2001 183 185 191 10.1086/317922 11120924

[B56] Miele G Seeger H Marino D Eberhard R Heikenwalder M Stoeck K Basagni M Knight R Green A Chianini F Urinary alpha1-antichymotrypsin: a biomarker of prion infection PLoS One 2008 3 e3870 10.1371/journal.pone.0003870 19057641 PMC2586086

